# Investigating the thermodynamic optimization of naturally convective flow in a corrugated enclosure: The influence of gap spacing and orientation of split baffles

**DOI:** 10.1016/j.heliyon.2024.e34930

**Published:** 2024-07-20

**Authors:** Asad Ali, Rashid Ali, Ahmed S. Hendy, Ali Altalbe

**Affiliations:** a School of Mathematics and Statistics, Central South University, Changsha, 410083, PR China; b School of Mathematical Sciences, Zhejiang Normal University, Jinhua, Zhejiang, 321004, PR China; c Department of Computational Mathematics and Computer Science, Institute of Natural Sciences and Mathematics, Ural Federal University, 19 Mira St., Yekaterinburg, 620002, Russia; d Department of Mechanics and Mathematics, Western Caspian University, Baku, 1001, Azerbaijan; e Department of Computer Science, Prince Sattam Bin Abdulaziz University, Al-Kharj, 11942, Saudi Arabia; f Faculty of Computing and Information Technology, King Abdulaziz University, Jeddah, 21589, Saudi Arabia

**Keywords:** Natural convection, Star corrugated cavity, Split baffle, Finite element method

## Abstract

The natural convection in cavities is frequently used in fluid mechanics and heat transfer engineering, such as heat exchangers, electronics, solar collectors, and growing crystals. However, the physics of natural convection flow and heat transfer in cavities with split baffles is least understood. The fundamental aim of this research is to investigate the impact of heated split baffles positioned at various locations on steady-state free convection within a sinusoidal corrugated star cavity. In this model configuration, the outer wavy enclosure is maintained at a constant temperature of ${\;T}_{c}$, while the inner split baffles are heated at a constant temperature of $T_{h}$. The finite element method is employed to discretize and solve the governing equations describing the fluid flow and heat transfer within the enclosure. This numerical approach aimed to analyze the effects of baffle inclination angles, baffle spacing, Rayleigh number on the fluid dynamics and convective thermal transport characteristics. The variation in velocity and temperature profile is illustrated through the streamlines and isotherm contours. Moreover, the numerical result is displayed in term ${Nu}_{avg}$ of the heat transfer, which are analyzed for inside space of baffles and angles of the baffle $\left(\theta =0^{0},45^{0},90^{0}\right)$. The key finding demonstrates that increasing the Rayleigh numbers and the different positions (up, central, down) of inner vertical split baffles enhances the magnitude of the velocity by 88.1%, 85.9 and 89.6% respectively. Furthermore, for the inner rectangular split baffle angles of 0°,45°, and 90°, and within the Rayleigh number range of $10^{4}$ to $10^{6}$, the ${Nu}_{avg}$ exhibits significant variations, with maximum increases of 71.9% , 46.7% and a subsequent decrease of 45.9%.

## 1 Introduction

The phenomenon of natural convection thermal transport in the chamber has been investigated in recent decades, especially during the rapidly developing electronic components industry. This natural convection setup is prevalent in various practical applications, including heat exchangers, electronics or LED cooling heat sinks, solar collectors, crystal growth, etc. The geometry with obstacles in the cavity is discussed by Mahdi et al. in Ref. [1]. Roy and Bask [2] investigated free convection in a corrugated enclosure with a warmed thin rectangle plate. Atayilmaz and Teke [3] used finite elements to analyze free convection flows within a square cage featuring non-uniformly warmed walls. Reymond et al. [4] investigated natural flow around a heated horizontal surface through numerical simulation and experimental methods. Esam and Alawadhi [5] discussed that natural convection facilitates the transfer of heat between the two horizontally oriented cylinders. Bhowmik and Tou [6] presented the convectional flow in a horizontal ring with periodically fluctuating interior cylinders, which is investigated using Lagrangian and Eulerian kinematics. Bocu and Altac [7] investigated thermal transport and entropy generation in a chamber stuffed with nanofluid under the impact of a Hartmann field computed by neural networks. Heindel et al. [8] explored the experimental examination of the transient free convection heat transport from simulated electronic circuits. Khalili et al. [9] studied the effects of laminar unforced convection on thermal transport and airflow in three-dimensional rectangular cages with fixed arrays attached to the hot wall. Sheikhzadeh et al. [10] analyzed the conversion of the flow of air caused by free convection from several separate heat sources. Ho et al. [11] examined the free convection of an Al2O3 nanofluid between two horizontal cylinders bounded by a circle. Dai et al. [12] explored the effect of shape on magneto-convection in a square container with a low Prandtl number. Adlam [13] studied the convection caused by the atmosphere of the Earth within two horizontal containers having an adiabatic spherical boundary. Park and Change [14] discussed a cylinder container and free convection among both warm and cool microtubes. Fujii et al. [15] analyzed time-dependent free convection within a chamber containing interior bodies in a two-dimensional. Chowdhury et al. [16] investigated turbulent nanofluid flow in a channel with triangular vortex generators computed by using artificial neural networks. Saglam et al. [17] showed a computational analysis of the interaction of laminar free convection generated between a pair of vertically spaced horizontal containers. Altaee et al. [18] examined the natural air convection from a collection of vertical parallel plates with distinct and projecting heat sources. Oztop and Abu-Nada [19] investigated unforced convection in a permeable triangle cage with a circular obstruction under thermal generation. Radhwan et al. [20] studied free convection in a chamber with two separate heat sources. Yesiloz and Aydin [21] discussed the unforced convection inside of a square enclosure, which holds an equilateral triangle in various orientations. Al-Zuhairy et al. [22] conducted a numerical investigation on free convection in rectangular, substantially warmed, nanofluid-filled spaces. Al-jabair and Habeeb [23] analyzed laminar free convection in a square container with distinct perpendicular wall warming. Khalili et al. [24] investigated free convection in a quadrantal inclined chamber with adjacent walls heated and cooled. Santra et al. [25] explored a numerical simulation of the transfer of heat phenomena within a constrained square chamber. Elatar et al. [26] modeled free convection in concentric annuli, featuring an inclined square chamber on the outer side and a horizontal cylinder on the inner side. Nakhi and Chamka [27] studied a square cavity employed in an experiment to examine the distribution of nanocrystals in the free convection of Al2O3-water nanofluid. Shi et al. [28] used the L-shaped cavity to simulate the transport of the red blood cell by FEM. Sahib et al. [29] examined the use of a copper-water nanofluid to improve heat transmission in a square hole that is being heated differentially. Sheikhzadeh et al. [30] analyzed laminar free convection in a rectangular container with a single horizontal fin. Oztop et al. [31] studied the effect of a thin fin's width and tilt on free convection in a square container. Jabbar et al. [32] presented that a thin fin on the wall of a differentially warmed square chamber causes laminar unforced convection, which is the process by which heat is transferred. Sankar et al. [33] investigated the unforced convection process within a nanofluid-filled square chamber, taking into account the influence of interior entities. Nardini et al. [34] studied the nanofluid naturally convection in a hollow with partially active side walls. Bilgen [35] analyzed the influence of non-isothermal variation in temperature on free convection in nanofluid-filled cages and investigated it using computational methods. Bhattacharya and Das [36] used CFD to evaluate unforced convection in a square cavity with two narrow baffles of different lengths and placements on the hollow's vertical walls on opposing sides. Asif et al. [37] presented the impact of a thin baffle's size and placement on free convection in a vertical annular enclosure. Saha and Gu [38] studied free convection in a square hollow with two baffles on the vertical walls, which was investigated experimentally and numerically. Varol and Oztop [39] proposed natural convection in buildings with part-wall enclosures. Holtzman et al. [40] studied the constant natural convectional heat transfer in a square chamber for various Rayleigh and Nusselt number values. Kandaswamy et al. [41] analyzed the heat transfers within an enclosure that is rectangular and has baffles. Ghalambaz et al. [42] studied the analysis focusing on the unforced convection of a suspension containing nano-encapsulated phase change materials within a permeable medium. Mehryan et al. [43] discussed a numerical study employing the Arbitrary Lagrangian-Eulerian (ALE) approach to investigate the unsteady unforced convection occurring within an inclined square chamber divided by a flexible impermeable membrane. Ghalambaz et al. [44] examined the free convection phenomenon and studied the fluid-structure interaction within a square chamber partitioned by a flexible membrane and subjected to sinusoidal temperature heating. Zadeh et al. [45] investigated the natural convection induced by a warmed plate within a chamber, focusing on elucidating the impact of plate flexibility on fluid flow and thermal transport characteristics. Ghalambaz et al. [46] analyzed the convective thermal transport and creation of entropy within a chamber containing a suspension of nano-enhanced Phase Change Materials (NEPCM). Furthermore, comprehensive research in Refs. [[47], [48], [49], [50]] aimed to simulate and analyze a nanofluid's free convection thermal transport within a quarter circle and a three-dimensional enclosure. They employed the lattice Boltzmann and mEDAM approaches, the graphical results validated the numerical simulations, and the obtained results were illustrated graphically.

In this paper, the main objective is to analyze three instances of (Ra) involving a thermal transport issue within a star chamber with rectangular split baffles. The impacts of the split baffle with different angles and positions on the fluid flow characteristics and heat transfer rate are simulated. Further, this work determines the best way to install the plate arrays in the cavity to enhance free convection thermal transport and provides a practical reference for engineering applications.

**Novelty:** The novelty of the present work is to fill the research gap by analyzing natural convection within a star-corrugated enclosure with split baffles. The study focuses on exploring the influence of surface roughness on natural convection due to the unique configuration of the corrugated cavity and assessing the effectiveness of split baffles. It also investigates the novel aspect of the interior space of the baffles and the variation of angles $\left(\theta =0^{0}-90^{0}\right)$ within the enclosure, which could potentially impact convective heat transfer. The Finite Element Method (FEM) is employed for numerical calculations, and flow visualization results are offered as isotherms and streamlines contours. Additionally, average Nusselt numbers for the hot walls were calculated and examined numerically.

**Fig. 1:**
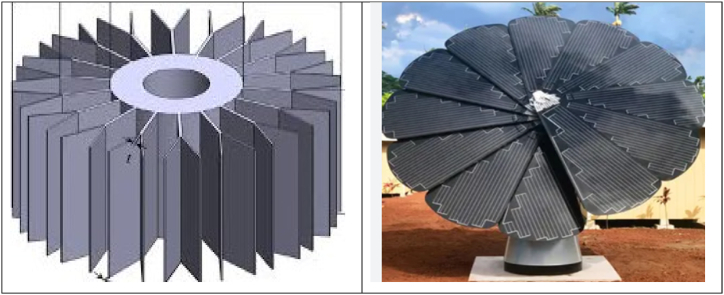
Physical application of the star corrugated cavity in the form of Solar Collector and Heat exchanger devices.

**Applications:** The mathematical model of a star corrugated cavity with split baffles has several physical applications (see Fig. 1). Here are some examples:

**Heat exchanger:** The geometry of the star corrugated cavity with split baffles has practical applications in heat exchanger design. It can enhance convection to improve efficiency across various sectors, such as HVAC, automotive cooling, and industrial heat recovery.

**Electronics cooling:** The model has the potential to be applied to cooling systems of heat exchangers, electronics cooling, thermal management in buildings, and renewable energy systems.

**Wave energy converter:** Corrugated surfaces may be explored in wave energy conversion technology, utilizing the dynamic movement of water to generate electricity in a sustainable and renewable approach.

**Solar Collectors:** The design with star-shaped baffles can enhance solar collector performance by improving heat transfer and fluid flow, resulting in increased thermal efficiency.

The rest of the paper is distributed as follows: In section 2, we discussed the problem description. In section 3, we formulated the mathematical model of our physical problem. In section 4, we discretized the mathematical model by the finite element procedure. In section 5, we illustrated some numerical experiments to analyze our proposed study. In the last section, some conclusion remarks are presented to conclude the impacts of our research work.

## 2 Problem description

**Fig. 2:**
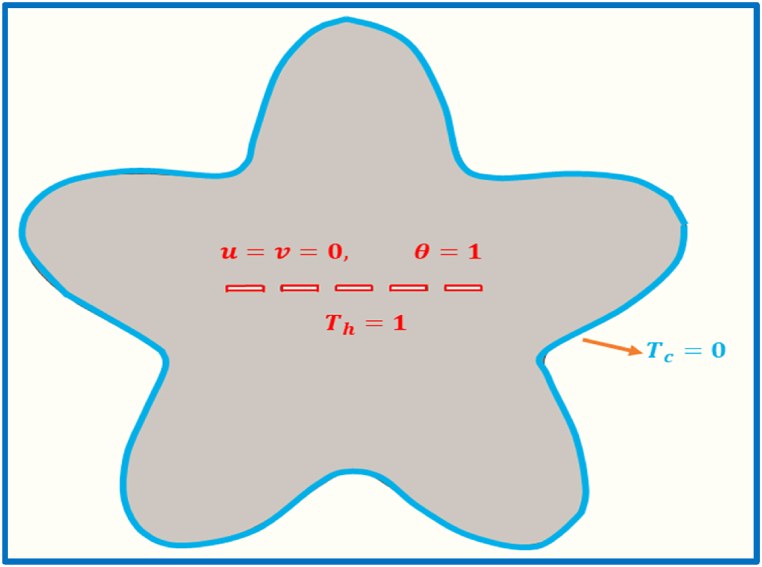
Schematic diagram of the physical model**.**

In this problem, we discussed the natural heat convection in a star-shaped corrugated cavity with rectangular split baffle. The split baffle is heated with temperature ($T_{h}$), whereas the enclosure's exterior walls are set to be cold with temperature ($T_{c}$). The boundary conditions for velocity on its surface are considered to be non-slip. The fluid with Prandtl number (Pr=7.0) is considered in the star corrugated enclosure. We assume that the fluid has constant density (i.e., the fluid is incompressible). The fluid in the aforementioned cavity dependent on temperature and can compute the changes in temperature and the fluid is independent of pressure changes. In this work, we insert the rectangular split baffle in the positions as up, center, and down in the cavity. The Rayleigh number parameter is presented ($10^{4}-10^{6})$ for the solution of the proposed partial differential equations. The problem is assumed to be in a steady state with laminar flow. The Boussinesq approximation is used to formulate the mathematical model which induces the natural convection term in the flow. The physical description of the anticipated physical domain of the star corrugated enclosure having inner split baffle is presented in Fig. 2.

## 3 Mathematical formulation

In the current mathematical model, the thermo physical properties, density fluctuations, and body forces are presented in the term of momentum equation. The Boussinesq approximation is employed to describe fluid characteristics, establishing a relationship between density changes with temperature and the coupling of the temperature field to the flow field. The governing equations for the steady state free convection flow employing energy, momentum, and mass equations are as follows [53,54]:

(1) $$\frac{\partial u}{\partial x}+\frac{\partial v}{\partial y}=0\;\text{.}$$

(2) $$u\frac{\partial u}{\partial x}+v\frac{\partial u}{\partial y}=-\frac{1}{\rho }\frac{\partial P}{\partial x}+v\left(\frac{\partial^{2}u}{\partial x^{2}}+\frac{\partial^{2}u}{\partial y^{2}}\right)\;\text{.}$$

(3) $$u\frac{\partial v}{\partial x}+v\frac{\partial v}{\partial y}=-\frac{1}{\rho }\frac{\partial P}{\partial x}+v\left(\frac{\partial^{2}v}{\partial x^{2}}+\frac{\partial^{2}v}{\partial y^{2}}\right)+g\beta \left(T-T_{c}\right)\;\text{.}$$

(4) $$u\frac{\partial v}{\partial x}+v\frac{\partial v}{\partial y}=\alpha \left(\frac{\partial^{2}T}{\partial x^{2}}+\frac{\partial^{2}T}{\partial y^{2}}\right)\;\text{.}$$

The associated boundary conditions for (1)−(4) are:

(5) $$\begin{array}{c} u=v=0,T=T_{h}=1\;\left(\text{Inner}\;\text{boundraies}\right)\\ u=v=0,T=T_{c}=0\;\left(\text{Outer}\;\text{boundraies}\right) \end{array}\;\}\text{.}$$

Where the x and y are the lengths evaluated horizontally and vertically, respectively. The velocity ingredients along the x and y directions are indicated as u and v, respectively. T represents the temperature; v and α represent kinematic resistance and thermal conductivity, respectively; p and ρ represent pressure respectively. The $T_{h}$ and $T_{c}$ are the temperatures at the hot and cold walls, respectively.

Using the following non-dimensional variables [55,56]:

(6) $$\begin{array}{c} X=\frac{x}{L},\\ Y=\frac{y}{L}\text{,}\\ U=\frac{uL}{\alpha }\text{,}\\ v=\frac{vL}{\alpha }\text{,}\\ P=\frac{pL^{2}}{\rho \alpha^{2}}\text{,}\\ \theta =\frac{T-T_{c}}{T_{h}-T_{c}} \end{array}\}\;\text{.}$$

Using the non-dimensional parameters defined in equation (6), the dimensional governing equations (1), (2), (3), (4), (5) can be converted into their respective dimensionless representations [53,54]:

(7) $$\frac{\partial U}{\partial X}+\frac{\partial V}{\partial Y}=0\;\text{.}$$

(8) $$U\frac{\partial U}{\partial X}+V\frac{\partial U}{\partial Y}=-\frac{1}{\rho }\frac{\partial P}{\partial X}+\mathrm{Pr}\;\left(\frac{\partial^{2}U}{\partial X^{2}}+\frac{\partial^{2}U}{\partial Y^{2}}\right)\;\text{.}$$

(9) $$U\frac{\partial V}{\partial X}+V\frac{\partial V}{\partial Y}=-\frac{1}{\rho }\frac{\partial P}{\partial Y}+\mathrm{Pr}\left(\frac{\partial^{2}V}{\partial X^{2}}+\frac{\partial^{2}V}{\partial Y^{2}}\right)+RaPr\theta \;\text{.}$$

(10) $$U\frac{\partial \theta }{\partial X}+V\frac{\partial \theta }{\partial Y}=\frac{\partial^{2}\theta }{\partial X^{2}}+\frac{\partial^{2}\theta }{\partial Y^{2}}\;\text{.}$$

With the non-dimensional boundary conditions,

(11) $$\begin{array}{c} U=0,V=0,\theta =1\;\left(\text{Inner}\;\text{boundraies}\right)\\ U=0,V=0,\theta =0\;\left(\text{Outer}\;\text{boundraies}\right) \end{array}\}\;\text{.}$$

Dimensionless coordinates X and Y fluctuate along the horizontal and vertical axes. U and V depicts dimensionless velocity ingredients in the X and Y directions; while θ represents temperature; P is stand for pressure; and Ra and Pr are Rayleigh and Prandtl numbers, respectively. The transfer of heat coefficient is described in terms of the local Nusselt number (${Nu}_{Local}$) and the mean Nusselt number (${Nu}_{Avg}$), as follows:

(12) $${Nu}_{Local}=-\frac{\partial \theta }{\partial n},{Nu}_{Avg}=\frac{1}{S}\;\int_{0}^{S}{Nu}_{Local}\;dS\;\text{.}$$

where n represents the normal direction on the plate.

## 4 Discretization by finite element method

### 4.1 Galerkin procedure

The Galerkin finite element procedure is used to solve the equations (8−10) of the momentum and energy equations. The continuity equation (7) is utilized as a constraint to generate the pressure distribution due to the mass conservation [2]. We utilized the penalty finite element method to solve equations (9)−(11), where the pressure P is reduced by an extra parameter and the incompressibility constraints specified by equation (7) (see Reddy [2]) which generates as follows,

(13) $$P=-\gamma \left(\frac{\partial U}{\partial X}+\frac{\partial V}{\partial Y}\right)\;\text{.}$$

The continuity equation (7) is obviously valid for the big values of γ. The prevalent values of γ produce a reliable result is $10^{7}$ [2]. By employing Eq. (13), the momentum dimensionless equations (9) and (10) are reduced as follows,

(14) $$U\frac{\partial U}{\partial X}+V\frac{\partial U}{\partial Y}=\gamma \frac{\partial }{\partial X}\left(\frac{\partial U}{\partial X}+\frac{\partial V}{\partial Y}\right)+\mathrm{Pr}\left(\frac{\partial^{2}U}{\partial X^{2}}+\frac{\partial^{2}U}{\partial Y^{2}}\right)\text{.}$$

(15) $$U\frac{\partial V}{\partial X}+V\frac{\partial V}{\partial Y}=\gamma \frac{\partial }{\partial Y}\left(\frac{\partial U}{\partial X}+\frac{\partial V}{\partial Y}\right)+\mathrm{Pr}\left(\frac{\partial^{2}V}{\partial X^{2}}+\frac{\partial^{2}V}{\partial Y^{2}}\right)+\text{Ra}\;\mathrm{Pr}\;\theta \text{.}$$

The velocity ingredients (U, V) and temperature (θ) are expressed by employing basis set ${\left\{\emptyset_{k}\right\}}_{k=1}^{N}$ as follows:

(16) $$U\approx \sum_{k=1}^{N}U_{k}\Phi_{k}\left(X,Y\right),V\approx \sum_{k=1}^{N}V_{k}\Phi_{k}\left(X,Y\right),\theta \approx \sum_{k=1}^{N}\theta_{k}\Phi_{k}\left(X,Y\right)\;\text{.}$$

For

0≤X,Y≤1,

The nonlinear residual equations for Eq. (14), (15), (16), and (10) were generated by the Galerkin finite element method at the nodes within the internal domain. Ω:

(17) $$R_{i}^{1}=\sum_{k}^{N}U_{k}\int_{\Omega }\left[\left(\sum_{k=1}^{N}U_{k}\Phi_{k}\right)\frac{\partial \Phi_{k}}{\partial X}+\left(\sum_{k=1}^{N}V_{k}\Phi_{k}\right)\frac{\partial \Phi_{k}}{\partial Y}\right]\Phi_{i}\text{dXdY}+\gamma \left[\sum_{k}^{N}U_{k}\int_{\Omega }\frac{\partial \Phi_{i}}{\partial X}\frac{\partial \Phi_{k}}{\partial X}\text{dXdY}+\sum_{k}^{N}V_{k}\int_{\Omega }\frac{\partial \Phi_{i}}{\partial X}\frac{\partial \Phi_{k}}{\partial X}\text{dXdY}\right]+\mathrm{Pr}\sum_{k}^{N}U_{k}\int_{\Omega }\left[\left[\frac{\partial \Phi_{i}}{\partial X}\frac{\partial \Phi_{k}}{\partial X}+\frac{\partial \Phi_{i}}{\partial Y}\frac{\partial \Phi_{k}}{\partial Y}\right]\text{dXdY}\right]\}\;\text{.}$$

(18) $$R_{i}^{2}=\sum_{k}^{N}V_{k}\int_{\Omega }\left[\left(\sum_{k=1}^{N}U_{k}\Phi_{k}\right)\frac{\partial \Phi_{k}}{\partial X}+\left(\sum_{k=1}^{N}V_{k}\Phi_{k}\right)\frac{\partial \Phi_{k}}{\partial Y}\right]\Phi_{i}\text{dXdY}+\gamma \left[\sum_{k}^{N}U_{k}\int_{\Omega }\frac{\partial \Phi_{i}}{\partial X}\frac{\partial \Phi_{k}}{\partial X}\text{dXdY}+\sum_{k}^{N}V_{k}\int_{\Omega }\frac{\partial \Phi_{i}}{\partial X}\frac{\partial \Phi_{k}}{\partial X}\text{dXdY}\right]+\mathrm{Pr}\sum_{k}^{N}V_{k}\int_{\Omega }\left[\left[\frac{\partial \Phi_{i}}{\partial X}\frac{\partial \Phi_{k}}{\partial X}+\frac{\partial \Phi_{i}}{\partial Y}\frac{\partial \Phi_{k}}{\partial Y}\right]\text{dXdY}\right]-RaPr\;\int_{\Omega }\left(\sum_{k=1}^{N}\theta_{k}\Phi_{k}\right)\;\Phi_{i}\text{dXdY}\}\;\text{.}$$

(19) $$R_{i}^{3}=\sum_{k}^{N}\theta_{k}\int_{\Omega }\left[\left(\sum_{k=1}^{N}U_{k}\Phi_{k}\right)\frac{\partial \Phi_{k}}{\partial X}+\left(\sum_{k=1}^{N}V_{k}\Phi_{k}\right)\frac{\partial \Phi_{k}}{\partial Y}\right]\Phi_{i}\text{dXdY}+\sum_{k}^{N}\theta_{k}\int_{\Omega }\left[\left[\frac{\partial \Phi_{i}}{\partial X}\frac{\partial \Phi_{k}}{\partial X}+\frac{\partial \Phi_{i}}{\partial Y}\frac{\partial \Phi_{k}}{\partial Y}\right]\text{dXdY}\right]\}\;\text{.}$$

The integrals in the residual equations are evaluated by using Gaussian quadrature for the three-point bi-quadratic basis functions. The second term carrying the penalty parameter (γ) is assessed with two points in Eqs. (17), (18). Eq. (18)− (19) are residuals which are represented in the matrix-vector representation by finite element procedure as follows:

(20) $$\left(K_{1}+\gamma K_{2}\;\right)a=F\text{.}$$

$K_{1}$, $K_{2}$ are the matrices derived from the Jacobian of the residuals where an indicates the indeterminate vector. The constraint equation is well satisfied, as γ occurs to a significant plenty ($∼10^{7}$). The result of the extension of the $K_{1}$ is negligible (use Eq. (20)) when compared to $\gamma K_{2}$**.**

(21) $${\;K}_{2}\alpha =\frac{F}{\gamma }\text{.}$$

This means that as γ approaches infinity, the governing equations are reduced to just the constraint condition, which is the continuity equation. Furthermore, because $K_{2}$ is nonsingular for large γ, the solution obtained from Eq. (21) is trivial. To achieve non-trivial solutions for large γ ($∼10^{7}$), $K_{2}$ must be a singular matrix. This is accomplished by applying two-point Gaussian quadrature for $K_{2}$ and three-point Gaussian quadrature for $K_{1}$.

The Newton-Raphson method is applied for solving the nonlinear residual equation (17) − (19) in order to ascertain the expansion coefficient in Eq. (16). The linear (3 N × 3 N) system is illustrated at each iteration.

(22) $$J\left(a^{n}\right)\;\left[a^{n}-a^{n+1}\right]=R\left(a^{n}\right)\text{,}$$

where n denotes the number of iterations index. The Jacobian matrix element $J\left(a^{n}\right)$ contains the velocity component derivatives of the residual equations, and the residual vector is represented by $R\left(a^{n}\right)$. Convergence criteria are used to determine the point at which the iterative process should end ${\left[\sum {\left(R_{i}^{\left(j\right)}\right)}^{2}\right]}^{\frac{1}{2}}\leq 10^{-5}$.

The iso-parametric mapping technique uses a nine-node bi-quadratic element in the two-dimensional X−Y plane. It is based on a unit square domain in the ξ−η plane and incorporates nine-node bi-quadratic basis functions to enhance the analysis of internal domain features in the residual equations.

(23) $$X=\sum_{i=1}^{9}X_{i}\Phi_{i}\;\left(\eta ,\xi \right)\;and\;Y=\sum_{i=1}^{9}Y_{i}\Phi_{i}\;\left(\eta ,\xi \right)\text{,}$$

The local bi-quadratic basis functions $\Phi_{i}\;\left(\eta ,\xi \right)$ are defined over the ξ−η domain. The integrals in Eqs. (17), (18), (19) can be computed within this domain using the following relationships:

(24) $$\left(\begin{array}{c} \frac{\partial \Phi_{i}}{\partial X}\\ \frac{\partial \Phi_{i}}{\partial Y} \end{array}\right)=\frac{1}{J}\left[\begin{array}{c} \frac{\partial Y}{\partial \eta }\;-\frac{\partial Y}{\partial \xi \;}\\ \frac{\partial X}{\partial \eta }\;-\frac{\partial X}{\partial \xi \;} \end{array}\right]\;\left[\begin{array}{c} \frac{\partial \Psi_{j}}{\partial \eta }\\ \frac{\partial \Psi_{j}}{\partial \xi } \end{array}\right],\text{here}\;dXdY=Jd\xi \;d\eta \text{.}$$

Where $J=\left|\frac{\partial \left(X,Y\right)}{\partial \left(\xi ,\eta \right)}\right|$. The local Nusselt number on a surface (${Nu}_{Local}$), defined in Eq. (12), utilizes the bi-quadratic basis set in the ξ−η domain for evaluating the normal derivative using Eqs. (23), (24).

### 4.2 Evaluation of stream function

The flow potential ψ are created from the velocity ingredients V and U, which is employed to convey the flow dynamic. For the two-dimensional flow, the connection between flow potential, ψ and velocity ingredients are as follows [57]:

(25) $$U=\frac{\partial \psi }{\partial Y},V=\frac{\partial \psi }{\partial X}\;\text{.}$$

That produce a singular equation [57]:

(26) $$\frac{\partial^{2}\psi }{\partial X^{2}}+\frac{\partial^{2}\psi }{\partial Y^{2}}=\frac{\partial U}{\partial Y}-\frac{\partial V}{\partial X}\;\text{.}$$

According to the description of the stream function given above, the positive sign of ψ indicates anti-clockwise circulation, while a negative value of ψ signifies clockwise circulation. The Galerkin finite element approach produces the subsequent linear residual equation for Eq. (26) through the expansion of the stream function (ψ) with the basis set ${\left\{\emptyset_{k}\right\}}_{k=1}^{N}$ as $=\sum_{k=1}^{N}\psi_{k}\Phi_{k}\left(X,Y\right)$ , and the incorporating the relationship for U, V from Eq. (16).

(27) $$R_{i}^{s}=\sum_{k}^{N}\psi_{k}\int_{\Omega }\left[\frac{\partial \Phi_{i}}{\partial X}\frac{\partial \Phi_{k}}{\partial X}+\frac{\partial \Phi_{i}}{\partial Y}\frac{\partial \Phi_{k}}{\partial Y}\right]\text{dXdY}+\sum_{k}^{N}U_{k}\int_{\Omega }\Phi_{i}\frac{\partial \Phi_{i}}{\partial Y}\;\text{dXdY}-\;\sum_{k}^{N}V_{k}\int_{\Omega }\Phi_{i}\frac{\partial \Phi_{i}}{\partial Y}\;\text{dXdY}\}\;\text{.}$$

Since there is no cross flow and the no-slip criteria holds true at all boundaries, ψ = 0 is employed as the residual equation at the boundary nodes. The bi-quadratic basis function is employed to compute the integrals in Eq. (27), and the temperatures ψ’s are determined by solving the N linear residual equations given in Eq. (27).

### 4.3 Validity of mesh generation

Grid independence testing is a crucial component of numerical experiments employing the finite element technique. The goal of grid independence testing is to identify the mesh size for a particular problem at which mesh refinement has little to no impact on the solution. To demonstrate the reliability of the capitalized computational system, the average Nusselt number is calculated using Pr=0.71 and $Ra=10^{6}$. Table 1 displays the statistics from this grid convergence test.

**Table 1:** Variation in ${Nu}_{avg}$ against different refinement levels.

*Refinement Level*	*No. of Elements*	*DOF*	$Nu_{Average}$
**R.L-01**	1210	6351	12.361
**R.L-02**	1386	7299	12.768
**R.L-03**	1716	8795	12.501
**R.L-04**	2859	14057	13.418
**R.L-05**	3947	18997	13.754
**R.L-06**	6301	29337	14.067
**R.L-07**	15049	67969	15.084
**R.L-08**	36606	159564	15.084
**R.L-09**	43702	188443	15.084

**Fig. 3:**
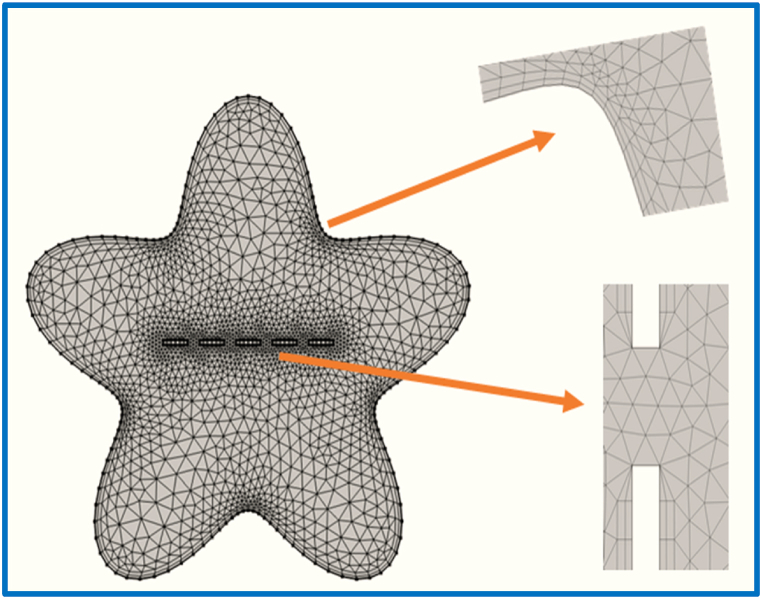
Grid refinement of the star cavity.

Fig. 3. Shows a more compact computational grid with triangular inside pieces and rectangular edges. While the number of elements and degree of freedom at various levels of refinement are shown in Table 1.

### 4.4 Validation of code

**Fig. 4:**
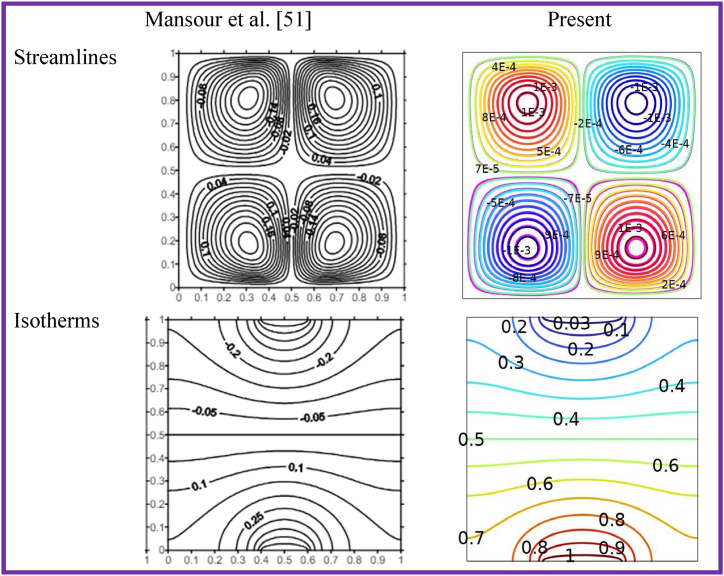
Comparison of streamlines and isotherms of current work with previous computational results of Mansour et al. [51].

To assess the reliability of the simulations conducted using COMSOL software, a verification test was conducted, wherein the results of the current study were compared to those of a previous study with certain limitations imposed. A comparison between the current isotherms and streamlines with the previous work computational results of Mansour et al. [51] at $Ra=10^{5}\;\text{and}\;\mathrm{Pr}=6.87$ is shown in Fig. 4. The current outcomes and those obtained by Mansour et al. [51] are in good agreement.

Additionally, Table 2 provides a comparison of the ${Nu}_{avg}$ for the case of the Hartmann field's impact on free convection in a corrugated enclosure filled with nanofluid. The number of Prandtl is fixed at 0.733, and the numbers of Rayleigh (Ra=Gr×Pr) are $1.466\times 10^{4}$ and $1.466\times 10^{5}$, respectively. The obtained outcomes for the ${Nu}_{avg}$ show good agreement with the results obtained by Afsana et al. [52], in which authors used the finite volume method (FVM) to solve the Navier-Stokes and energy equations.

**Table 2:** Comparing present results with Afsana et al. [52] for MHD effects on ${Nu}_{avg}$ where Pr=0.733..

Ra	Ha	Present	Afsana et al. [52]	(Error) %
$1.466\times 10^{4}$	0	2.5193	2.5098	−0.37
	10	2.2243	2.2203	−0.18
	50	1.1113	1.1105	−0.072
	100	1.0199	1.0187	−0.11
$1.466\times 10^{5}$	0	5.0363	5.0352	−0.021
	10	4.8374	4.8332	−0.086
	50	2.5985	2.5946	−0.15
	100	1.4298	1.4285	−0.091

## 5 Numerical experiments

In this section, we will compute several experiments to support our proposed study that how the heat is transferred naturally within a star enclosure with inner heated baffle via convection characterized by corrugated walls. Our main objective is to explore that how we can correlate the parameters to the impact of the temperature distributions and the intensity of streamlines. Additionally, we present viscous and thermal contour plots to facilitate a visual understanding of the natural convection patterns influenced by various factors. We employed a broad range of parameter variations, spanning from $Ra=10^{4}$ to $Ra=10^{6}$, while holding Pr constant at 0.71, to clarify the particular changes in associated profiles.

### 5.1 Effect of Ra on streamlines and isotherms with horizontal split baffles (UP, central, down)

**Fig. 5:**
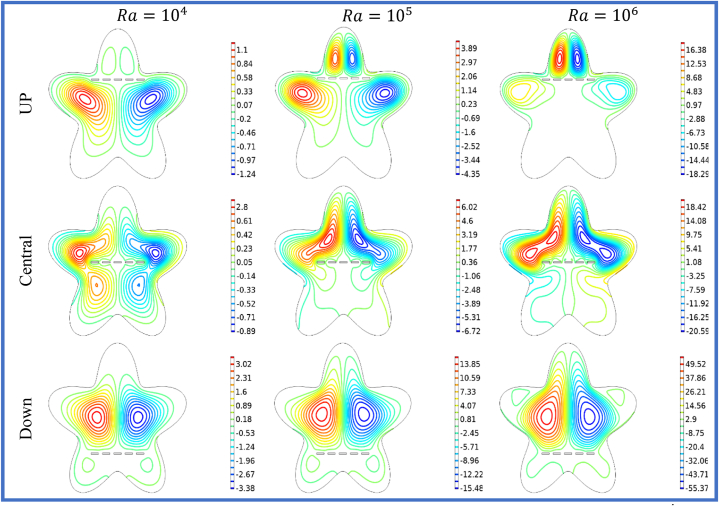
Variation of the streamlines with horizontal split baffles (UP, Central, Down) at $Ra=10^{4}-10^{6}$.

**Fig. 6:**
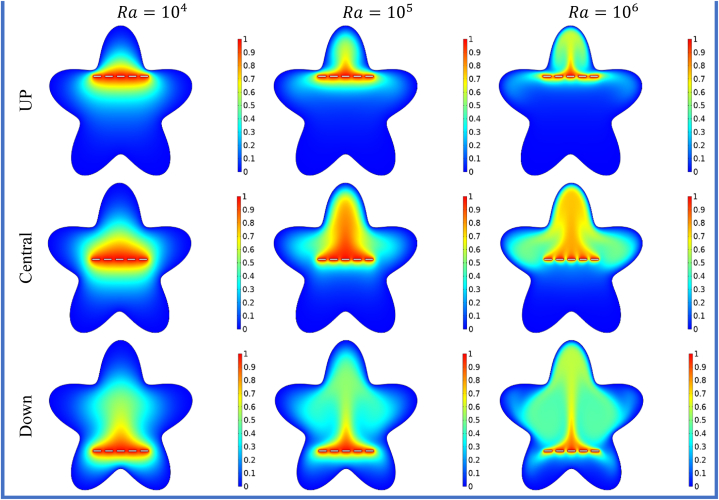
Variation of the isotherms with horizontal split baffles (UP, Central, Down) at $Ra=10^{4}-10^{6}$.

Fig. 5, Fig. 6 illustrates the impact of horizontal split baffles (up, central, down) and Rayleigh numbers (Ra) on the streamlines and isotherms. For $={\;10}^{4}$ ${-10}^{6}$, the maximum velocity magnitudes for horizontal split baffles (up, central, and down) are (1.1,3.89,16.38), (2.8,6.02,18.42) and (3.02,13.85,49.52) respectively. The presence of these baffles is evident in their disruption of the flow pattern and creation of additional flow paths, which in turn induce flow separation and reattachment. This disruption facilitates improved mixing and convective heat transfer. With increasing Rayleigh numbers, buoyancy forces become dominant, resulting in intensified convective currents and higher fluid velocities. The combination of these baffles and higher Rayleigh numbers synergistically enhances fluid motion, leading to an overall amplification in the velocity magnitude within the system. Fig. 6 depicted that with an increase in *Ra* from $10^{4}-10^{6}$, the impact of buoyancy forces becomes more prominent, leading to significant changes in the isotherm patterns. Combining the baffles and higher Rayleigh numbers results in complex and intricate temperature distributions, indicating enhanced convective heat transfer and thermal mixing. The change in heat flux co-efficient versus deviation in (Ra) and (S) is envisioned in Table 3 wide range of (Ra) varying from $10^{4}$ to $10^{6}$ is selected. The different cases of splitting are discussed in organized manner. In this table the result of splitting baffles is enumerated in comparative manner with no splitting (S=0) and with splitting space ($S_{1}-S_{3}=0.23-0.31$). It is noticed that by increasing the (Ra) and (S), the ${Nu}_{avg}$ will up 17.231 and 33.632.In view of impact of splitting of baffles will be enhanced the ${Nu}_{avg}$ will be escalate 48%to75% for the Up, Central and Down split baffle as shown in the Table 3, Table 4, Table 5 in details.

**Table 3:** The numerical variation in ${Nu}_{avg}$ against Ra for the up horizontal splitting baffle in a star cavity.

Ra	Horizontal case (center) (S)	Horizontal case1(Center) (S1)	Horizontal case2(Center) (S2)	Horizontal case3(Center) (S3)	% Between S and S3	% Between S1 and S3
$10^{4}$	5.964	6.245	9.712	18.608	67.9%	66.4%
$10^{5}$	9.977	10.548	15.996	24.231	59.6%	56.4%
$10^{6}$	17.231	21.232	26.264	33.632	48.7%	36.8%

**Table 4:** The numerical variation in ${Nu}_{avg}$ against Ra for the central horizontal splitting baffle in a star cavity.

Ra	Horizontal case Zero splitting (S)	Horizontal case Baffles space 0.23 (S1)	Horizontal case Baffles space 0.27 (S2)	Horizontal case Baffles space 0.31 (S3)	% Between S and S3	% Between S1 and S3
$10^{4}$	7.557	10.815	17.602	28.228	73.2%	61.6%
$10^{5}$	11.661	16.668	21.767	32.560	64.1%	48.8%
$10^{6}$	18.906	28.904	38.814	50.214	62.3%	42.4%

**Table 5:** The numerical variation in ${Nu}_{avg}$ against Ra for the down horizontal splitting baffle in a star cavity.

Ra	Horizontal case Zero splitting (S)	Horizontal Baffles with space 0.23 (S1)	Horizontal Baffles with space 0.27 (S2)	Horizontal Baffles space 0.31 (S3)	% Between S and S3	% Between S1 and S3
$10^{4}$	9.294	17.512	25.861	37.992	75.5 %	53.9%
$10^{5}$	13.282	22.712	30.994	43.938	69.7%	48.3%
$10^{6}$	25.322	37.513	47.819	62.355	59.3%	39.8%

**Table 6:** The numerical Variation in ${Nu}_{avg}$ against Ra for the up vertical splitting baffles in a star cavity.

Ra	VerticalCentral case (Q)	Vertical Central case (Q1)	Vertical Central case (Q2)	Vertical Central case (Q3)	% Between Q and Q3	% Between Q1 and Q3
$10^{4}$	32.925	22.543	17.443	10.268	68.8%	54.4%
$10^{5}$	46.959	38.423	28.321	18.101	61.4%	52.8%
$10^{6}$	62.602	48.481	37.481	26.812	57.1%	44.6%

**Table 7:** The numerical variation in ${Nu}_{avg}$ against Ra for the central vertical splitting baffle in a star cavity.

Ra	Vertical Case Zero splitting (Q)	Vertical Case Baffles space 0.215(Q1)	Vertical Case Baffles space 0.22 (Q2**)**	Vertical Case Baffles space 0.225(Q3)	**%** Between Q and Q3	% Between Q1 and Q3
$10^{4}$	47.976	37.724	27.525	20.218	57.8%	46.4%
$10^{5}$	65.854	50.701	38.565	28.246	57.1%	44.2%
$10^{6}$	74.243	60.997	48.307	37.145	49.9%	39.1%

**Table 8:** The numerical variation in ${Nu}_{avg}$ against Ra for the down vertical splitting baffle in a star cavity.

Ra	Vertical Case Zero splitting (Q)	Vertical Case Baffles space 0.215(Q1)	Vertical Case Baffles space 0.22(Q2)	Vertical Case Baffles space 0.225(Q3)	% Between Q and Q3	% Between Q1 and Q3
$10^{4}$	62.915	50.471	38.241	26.912	57.2%	46.6%
$10^{5}$	76.709	65.226	52.115	40.841	46.7%	37.3%
$10^{6}$	91.616	82.666	71.444	55.924	38.9%	32.3%

**Table 9:** The numerical variation in ${Nu}_{avg}$ against for the Up inclined with angle $45^{0}$ splitting baffle in a star cavity.

Ra	Inclined Central case (P)	Inclined Central case1 (P1)	Inclined Central case2 (P2)	Inclined Central case3 (P3)	% Between P and P3	% Between P1 and P3
$10^{4}$	6.915	8.224	18.951	28.744	74.9%	71.3%
$10^{5}$	11.909	13.541	23.821	34.861	65.8%	61.1%
$10^{6}$	20.616	22.995	33.415	45.211	54.4%	49.1%

### 5.2 Effect of Ra on streamlines and isotherms with vertical split baffles (UP, central, down)

**Fig. 7:**
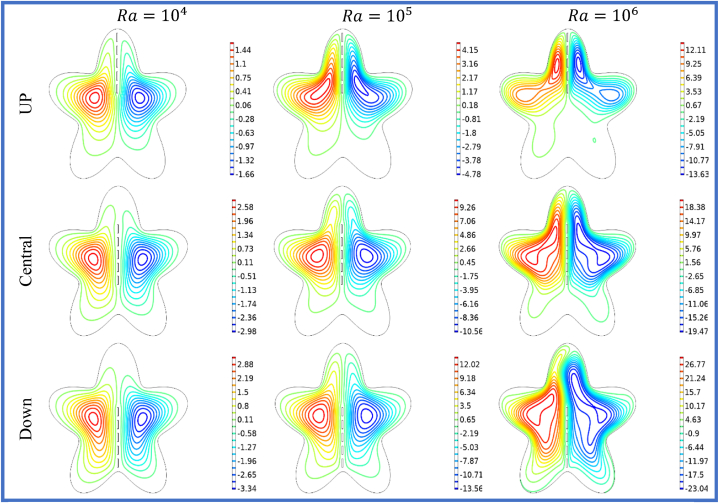
Variation of the streamlines with vertical split baffles (UP, Central, Down) at $Ra=10^{4}-10^{6}$.

**Fig. 8:**
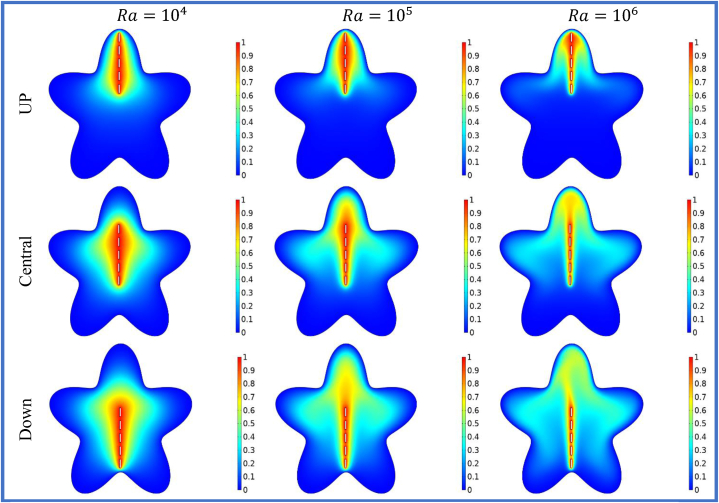
Variation of the isotherms with vertical split baffles (UP, Central, Down) at $Ra=10^{4}-10^{6}$.

Fig. 7, Fig. 8 depicts the streamlines and isotherms at $Ra=10^{4}-10^{6}$, $\theta =90^{0}$, and for different positions of split baffle (up, central, down). The heat transfer within the star cavity can be improved by the existence of vertically split baffle. By creating the vertical split baffle enhance the effective surface area available for the heat transmission and support the convective heat transfer. This result shows the better cavity cooling and heat dissipation phenomena. Moreover, the variation of the Rayleigh number effects the expanding of the vertical baffle spaces on the stream function and isothermal lines. The heat transfer rates and buoyancy-driven flows are high for the higher Rayleigh numbers. The effect of the split baffle on the flow and temperature distribution within the cavity become more noticeable as the Rayleigh number enhances as shown in the Figure 7 and 8. The Table 6, Table 7, Table 8 show that when the vertical splitting of baffle (up, central and down) is increasing then the ${Nu}_{avg}$ is decreasing significantly.

### 5.3 Effect of Ra on streamlines and isotherms with inclined $\left(45^{o}\right)$ split baffles (UP, central, down)

**Fig. 9:**
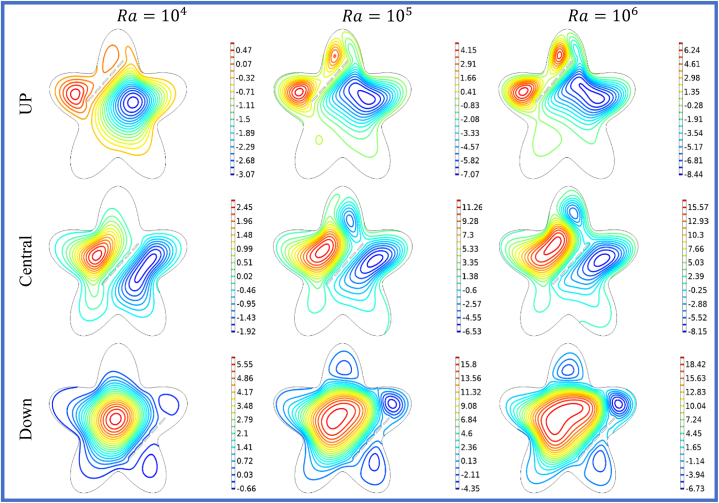
Variation of the streamlines with inclined ($45^{o})$ split baffles (UP, Central, Down) at $Ra=10^{4}-10^{6}$.

**Fig. 10:**
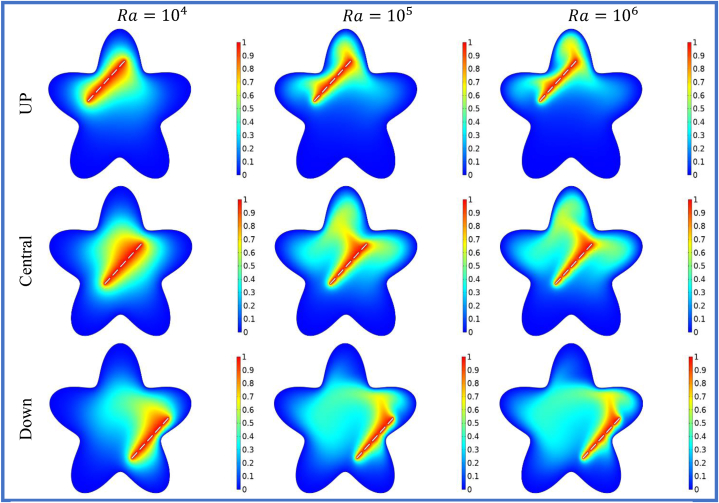
Variation of the isotherms with inclined ($45^{o})$ split baffles (UP, Central, Down) at $Ra=10^{4}-10^{6}$.

Fig. 9, Fig. 10 show the impact of different Rayleigh numbers (*Ra*) on streamlines and isotherms for inclined ($45^{0})$ inner rectangular split baffles (up, central, down). For $Ra=10^{4}-10^{6}$, the velocity magnitude increases by 92.4%, 84.2%, and 69.8% for the up, central, and down positions of the inclined split baffles, respectively. The inclined split baffles disturb the flow pattern, which helps to create turbulence and better mixing. When the Rayleigh numbers are higher, buoyancy forces become more dominant, strengthening the convective currents. As a result, the combination of inclined split baffles and increased Rayleigh numbers leads to clearer and more defined temperature differences shown by the isotherms within the corrugated enclosure.

For the inclined split baffle, the details are shown in the Table 9, Table 10, Table 11 the splitting of baffles increases, and then the average Nusselt number is enhanced.

**Table 10:** The numerical variation in ${Nu}_{avg}$ against for the central inclined with angle $45^{0}$ splitting baffle in a star cavity.

Ra	Inclined Case Zero splitting **(**P**)**	Inclined Case1 Baffles space 0.155 (P1)	Inclined Case2 Baffles space 0.175 (P2)	Inclined Case3 Baffles space 0.20 (P3)	% Between P and P3	% Between P1 and P3
$10^{4}$	10.325	17.936	25.197	36.135	71.4%	50.3%
$10^{5}$	15.459	21.534	32.682	48.649	68.2%	55.7%
$10^{6}$	26.602	33.321	42.978	55.189	51.7%	39.6%

**Table 11:** The numerical variation in ${Nu}_{avg}$ against Ra for the down inclined with angle $45^{0}$ split baffle in a star cavity.

Ra	**I**nclined Case Zero splitting (P)	Inclined Case1 Baffles space 0.155 **(**P1**)**	Inclined Case2 Baffles space 0.175 **(**P2**)**	Inclined Case3 Baffles space 0.20 **(**P3**)**	% Between P and P3	% Between P1 and P3
$10^{4}$	14.676	25.776	36.135	48.424	69.6%	46.7%
$10^{5}$	23.854	31.213	44.514	62.851	61.6%	50.3%
$10^{6}$	33.243	43.821	55.189	70.718	52.9%	38.1%

### 5.4 Average nusselt number

**Fig. 11:**
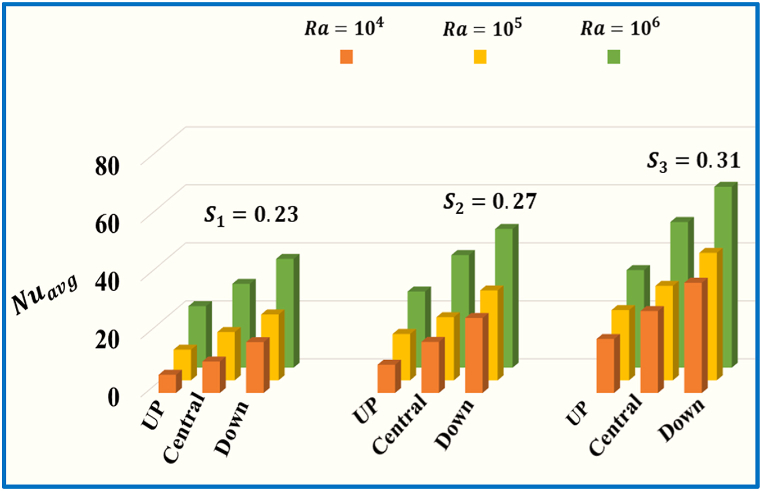
Variation of ${Nu}_{avg\;}$ for different space of horizontal split baffles (UP, Central, Down) against Ra.

**Fig. 12:**
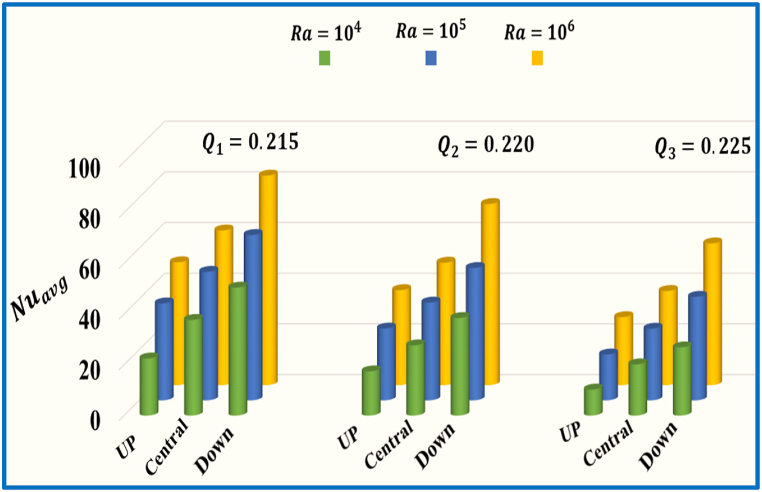
Variation of ${Nu}_{avg\;}$ for different space of vertical split baffles (UP, Central, Down) against Ra.

**Fig. 13:**
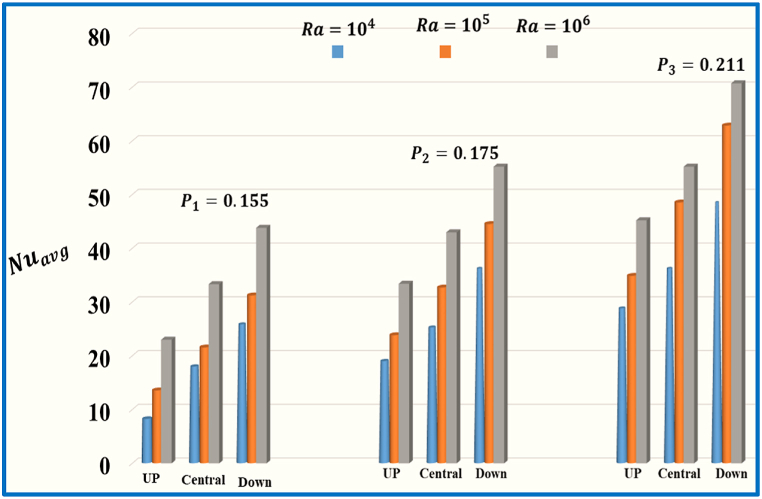
Variation of ${Nu}_{avg\;}$ for different space of inclined ($45^{o})$ split baffles (UP, Central, Down) against Ra.

Fig. 11 illustrates the influence of different spaces of horizontal split baffle (up, central and down) on the average Nusselt number against Ra with a fixed Pr=0.71. As the Rayleigh number (Ra) upsurge from $Ra=10^{4}$ to $10^{6}$, there is a notable growth in the values of the ${Nu}_{avg}$. Additionally, when the baffle spacing increases from 0.23 to o.31, the ${Nu}_{avg}$ concurrently increases, and indicating a stronger convective thermal transport effect. Fig. 12 represents the impact of number of Rayleigh $\left(Ra=10^{4},10^{5},10^{6}\right)$ and different spaces of vertical split baffle (up, central, down) on the ${Nu}_{avg}\text{.}$ As the baffle spacing increases (0.215 to 0.225), it leads to larger gaps between the baffles, allowing for more fluid flow and reduced obstruction. This results in decreased fluid mixing and reduced convective heat transfer, decline the ${Nu}_{avg}\text{.}$ Conversely, there is a significant escalate in the values of ${Nu}_{avg}\text{.}$ when Ra enhance from $10^{4}\text{,}$ to $10^{6}$. Fig. 13 comprehensively compares different values of inclined split baffle spaces and their corresponding ${Nu}_{avg}\text{.}$ , plotted against a range of Rayleigh numbers. As the inner inclined split baffle spacing increases from 0.155 to 0.211, fluid circulation is enhanced, convective heat transfer is increased, and the ${Nu}_{avg}$ is raised.

## 6 Conclusion

In this research, we studied the effect of the interior space of plates and the variation of the angles of the baffles on heat transfer in a corrugated star cavity. We also investigated the correlations between the Nusselt number and Rayleigh number in different baffle positions. The findings can be addressed to draw a conclusion and make remarks for our proposed study.

• Increasing the number of Rayleigh and different position of split baffles ($\theta =0^{o}-90^{o})$ enhances the strength of the fluid flow.
• The heat transfer mode of convection will be dominant when the Rayleigh numbers increase.
• The average Nusselt number (${Nu}_{avg}$) for various spaces of the inner horizontal split baffle increases by 67.4% when $S_{1}=0.23$, 71.9% when $S_{2}=0.27$, and 82.5.4% when $S_{3}=0.31$, at Rayleigh numbers (Ra) from $10^{4}$ to $10^{6}$.
• The ${Nu}_{avg}$ is directly proportional to the Ra and inversely proportional to the vertical increase in split baffle spaces.
• The average Nusselt number (${Nu}_{avg}$) progressively increases when the baffles are in inclined positions (center, up, and down) for the baffles spacing rises from 0.155 to 0.211..

**Table:** 

Nomenclature	
Ra	Rayleigh number
Pr	Prandtl number
${Nu}_{avg}$	Average Nusselt number
$T_{h}$	The heated surface thermal (K)
$T_{c}$	The cold surface thermal (K)
u,v	Dimensional velocity components
U,V	Dimensionless velocity components
S	Zero space in horizontal baffle
$S_{1},S_{2},S_{3}$	Horizontal baffles spacing
Q	Zero space in vertical baffle
$Q_{1},Q_{2},Q_{3}$	Vertical baffles spacing
P	Zero space in inclined baffle
$P_{1},P_{2},P_{3}$	Inclined baffles spacing
θ	Angle of split baffles
**Subscripts**	
c	Cold
h	Hot
avg	Average

## Ethics approval and consent to participate

Not applicable.

## Consent for publication

Not applicable.

## Data availability

Data will be made available on request.

## CRediT authorship contribution statement

**Asad Ali:** Writing – review & editing, Writing – original draft, Visualization, Validation, Software, Resources, Project administration, Methodology, Investigation, Funding acquisition, Formal analysis, Data curation, Conceptualization. **Rashid Ali:** Writing – review & editing, Writing – original draft, Supervision, Methodology, Formal analysis. **Ahmed S. Hendy:** Writing – review & editing, Writing – original draft, Resources, Investigation, Formal analysis. **Ali Altalbe:** Writing – review & editing, Writing – original draft, Project administration, Funding acquisition, Data curation.

## Declaration of competing interest

The authors declare that they have no known competing financial interests or personal relationships that could have appeared to influence the work reported in this paper.
